# Achievable Rate Optimization for Reconfigurable Intelligent Surface-Aided Multi-User Movable Antenna Systems

**DOI:** 10.3390/s25154694

**Published:** 2025-07-29

**Authors:** Liji Yu, Yuhui Ren

**Affiliations:** School of Information Science and Technology, Northwest University, Xi’an 710127, China

**Keywords:** antenna position optimization, beamforming, movable antenna, reconfigurable intelligent surface (RIS), sequential quadratic programming (SQP)

## Abstract

This paper proposes a novel optimization framework for reconfigurable intelligent surface (RIS)-aided movable antenna (MA) systems, tackling the joint optimization problem of beamforming and antenna positions. Unlike traditional approaches, we reformulate the antenna positioning task as a sequential quadratic programming (SQP) problem, enabling efficient handling of nonlinear spatial constraints through iteratively solved quadratic subproblems. An alternating optimization scheme is adopted to decouple the overall problem into two subproblems: (1) optimal beamforming using maximum ratio transmission (MRT) and fixed-point iteration, and (2) precise antenna location optimization via SQP. Simulation results demonstrate that the proposed method significantly enhances spectral efficiency by fully exploiting the synergistic benefits of RIS and MA technologies. The proposed method could achieve about a 25% performance improvement compared to the fixed-position scheme. Current approaches predominantly rely on gradient search methods, which fail to fully exploit the potential of positional DoFs. In contrast, our proposed method is more effective.

## 1. Introduction

With the ongoing evolution of wireless communications, increasing emphasis is being placed on leveraging spatial resources to enhance system capacity, reliability, and adaptability. A central theme in this pursuit is the active exploitation of the wireless propagation environment through intelligent design and control. Notably, several promising techniques have been developed to improve wireless communication performance, including millimeter-wave and terahertz communications [1,2], reconfigurable intelligent surface (RIS) [3,4], integrated sensing and communications (ISAC) [5,6], and near-field communications [7,8,9].

RIS is a revolutionary technology in wireless communication that offers dynamic control over electromagnetic wave propagation through passive, programmable metasurfaces [10,11]. By adjusting the phase and amplitude of reflected signals, RIS can enhance coverage, improve spectral efficiency, and reduce power consumption. A core capability is passive beamforming, whereby RIS intelligently directs signals toward intended users without active radio frequency (RF) components, enabling energy-efficient network optimization [12,13]. However, channel estimation remains a critical challenge due to the passive nature of RIS, and is often addressed via compressed sensing or machine learning techniques [14]. Recent advancements include double-RIS configurations to overcome line-of-sight blockages [15,16], multi-RIS systems for extended coverage [17,18,19], and extremely large-scale RIS for ultra-precise beamforming and near-field communication [20]. These innovations position RIS as a key enabler for 6G, supporting massive MIMO, smart radio environments, and IoT connectivity, although challenges such as real-time reconfiguration, hardware limitations, and scalability remain open research problems.

In addition to using RIS to enhance channel conditions, the concept of spatial reconfiguration via antenna repositioning has recently attracted growing interest. Movable antennas (MAs) provide the ability to dynamically adjust their physical locations, thereby influencing multipath propagation through geometric diversity [21]. Similarly, fluid antenna systems (FASs) adopt a comparable philosophy by enabling antenna movement within a confined region to optimize reception [22]. In [23], various practical implementations for MAs are discussed, such as electronic motor, liquid fluidity, and micro-electromechanical systems. These emerging paradigms extend traditional beamforming by incorporating geometric flexibility into system design, aligning with broader efforts to enhance environmental adaptability in wireless channels.

Motivated by these developments, the proposed MA-RIS system is particularly suited for scenarios with dynamically changing environments, such as urban canyons, high-speed vehicular networks, and indoor areas with frequent signal blockage. In such cases, the movable antenna (MA) array enables spatial reconfiguration to optimize the geometry of the transmission path, while the RIS compensates for severe path loss or non-line-of-sight (NLoS) conditions through intelligent signal reflection. This synergy allows the system to maintain high spectral efficiency and robust communication under a wide range of practical deployment scenarios.

### 1.1. Related Work

In [24], it was shown that spatial variations in MA locations induce periodic fluctuations in multipath channel gain, offering practical insights into spatial channel modeling. The study in [22] derived a closed-form lower bound on the capacity of FAS, revealing untapped potential within confined volumes. In [25], it was demonstrated that the achievable diversity of FAS scales with the number of ports under maximum ratio combining. A trade-off between achievable multiplexing gain and interference suppression was explored in [26], while user-side mobility was utilized in [27,28] to reduce transmit power. Moreover, MA-enhanced sum-rate maximization, transmit power minimization, and delay minimization were investigated in [29,30,31], further validating the potential of MA in improving communication performance. Beyond traditional MIMO applications, MA has been integrated with emerging paradigms such as ISAC [32,33] and RIS [34,35,36,37,38] to expand their capabilities.

The combination of RIS and MA technologies has demonstrated promising potential in recent works [34,35,36,37,38], as they synergistically enhance wireless channels. For instance, ref. [34] maximizes the sum rate using gradient-based methods to optimize the antenna position at the base station (BS). Similarly, ref. [35] investigated this scenario by using the generic algorithm for BS antenna position and RIS reflection phase optimization. Beyond exploring the degrees of freedom (DoFs) of the BS’s array geometry, refs. [36,38] investigate the performance analysis of array geometry adjustment at the RIS end. They demonstrate the significance of array geometry optimization of the RIS. Moreover, ref. [37] explores a single-antenna setup in which RIS element positions are optimized via Bayesian optimization to improve channel gain at the cost of increased complexity.

### 1.2. Contribution

While the existing literature has made significant advancements in RIS-MA systems, the additional DoFs introduced by movable antenna elements create substantial complexity in the joint optimization of antenna positioning, active beamforming, and passive beamforming. Furthermore, current approaches predominantly rely on gradient search methods, which fail to fully exploit the potential of positional DoFs. To overcome these limitations, our work makes the following key contributions:

- We model the RIS-aided multi-input single-output (MISO) downlink MA system and propose a novel and efficient joint optimization framework for it.
- We propose the fixed-point iteration (FPI) for the formulated problem in RIS-aided MA systems to efficiently solve the passive reflection phase, which can iteratively recover the phase at low complexity.
- We address an RIS-aided MA system by reformulating the BS antenna position optimization as an SQP problem. This formulation enables efficient handling of both nonlinearity and spatial constraints via iterative quadratic programming (QP). To jointly optimize RIS coefficients, beamforming, and antenna positions, we propose an alternating optimization framework that alternates between fixed-point iteration for RIS and beamforming, and SQP for antenna placement.

The rest of this paper is organized as follows: Section 2 provides the system model, Section 3 formulates the problem and presents the solution, Section 4 evaluates the proposed method via simulation, and Section 5 concludes this paper.

In this paper, vectors and matrices are denoted by boldface lowercase and uppercase letters (e.g., x and X), respectively. For any complex scalar *x* or vector x, we denote the conjugate by $x^{*}$, transpose by $x^{T}$, and conjugate transpose by $x^{H}$. The modulus and $\ell_{2}$-norm are denoted by |x| and ∥x∥, respectively. The expectation operator is E{·}, while Re{·} and Im{·} extract real and imaginary parts. Matrix operations include the trace Tr(·) and identity matrix $I_{N}$ of size N×N.

## 2. System Model

**Figure 1:**
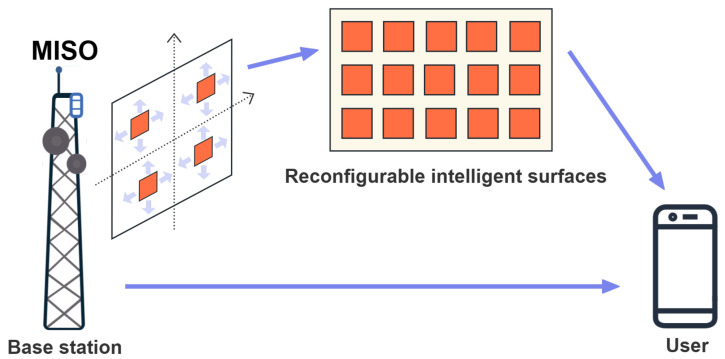
Illustration of the RIS-assisted MISO system.

We consider an RIS-assisted MISO downlink system, where the BS is equipped with *N* antennas, the RIS comprises *M* passive reflecting elements, and the user is equipped with a single antenna (see Figure 1). The planar arrays are deployed for the BS and RIS. The positions of the BS antennas are denoted by ${\left\{x_{n},z_{n}\right\}}_{n=1}^{N}$. The reflection coefficients of the RIS elements are given by ${\left\{e^{j\psi_{m}}\right\}}_{m=1}^{M}$, where $\psi_{m}$ denotes the reflection phase of the *m*-th RIS element. The RIS reflection matrix is defined as $\Phi =\mathrm{diag}\left(v\right)\in C^{M\times M}$, where $v={\left[e^{j\psi_{1}},\ldots ,e^{j\psi_{M}}\right]}^{T}$. For practical finite-sized passive RIS, the amplitude efficiency factor is typically less than 1. However, its normalized form is commonly adopted in the literature [4], where the amplitude is set to unity.

The signal received by the user can be expressed as

(1) $$y=h_{r}^{H}\Phi Gfs+h_{d}^{H}fs+n,$$

where $h_{r}\in C^{M\times 1}$ denotes the channel between the RIS and the user, $G\in C^{M\times N}$ represents the channel between the BS and the RIS, and $h_{d}\in C^{N\times 1}$ denotes the direct channel between the BS and the user. The vector $f\in C^{N\times 1}$ is the precoding vector, *s* is the transmitted symbol, and *n* is the additive noise, modeled as a circularly symmetric complex Gaussian (CSCG) random variable with distribution $\mathrm{CN}(0,\sigma^{2})$.

To characterize the spatial structure of the wireless channel, we consider a geometric channel model. Assuming that the channel G consists of *L* propagation paths, it is given by

(2) $$G=\sum_{l=1}^{L}\beta_{l}a_{R}\left(\phi_{l}^{\mathrm{ele}},\phi_{l}^{\mathrm{azi}}\right)a_{B}^{H}\left(\theta_{l}^{\mathrm{ele}},\theta_{l}^{\mathrm{azi}}\right),$$

where $\beta_{l}$ denotes the complex gain of the *l*-th propagation path, for l=1,…,L. The angle pairs $\left\{\phi_{l}^{\mathrm{ele}},\phi_{l}^{\mathrm{azi}}\right\}$ and $\left\{\theta_{l}^{\mathrm{ele}},\theta_{l}^{\mathrm{azi}}\right\}$ represent the elevation and azimuth angles of arrival (AoA) and departure (AoD), respectively. The vectors $a_{R}\in C^{M\times 1}$ and $a_{B}\in C^{N\times 1}$ denote the array response vectors at the RIS and the BS, respectively.

The channel between the RIS and the user is modeled as

(3) $$h_{r}=\sum_{p=1}^{P}\alpha_{p}a_{R}\left(\vartheta_{p}^{\mathrm{ele}},\vartheta_{p}^{\mathrm{azi}}\right),$$

where $h_{r}\in C^{M\times 1}$ represents the reflected channel vector. The summation accounts for *P* propagation paths, each associated with a complex gain $\alpha_{p}$. The array response vector $a_{R}\left(\vartheta_{p}^{\mathrm{ele}},\vartheta_{p}^{\mathrm{azi}}\right)$ corresponds to the RIS response to a signal arriving from the direction defined by the elevation angle $\vartheta_{p}^{\mathrm{ele}}$ and azimuth angle $\vartheta_{p}^{\mathrm{azi}}$ of the *p*-th path.

Similarly, the direct channel between the BS and the user is expressed as

(4) $$h_{d}=\sum_{k=1}^{K}\gamma_{k}a_{B}\left(\varphi_{k}^{\mathrm{ele}},\varphi_{k}^{\mathrm{azi}}\right),$$

where $h_{d}\in C^{N\times 1}$ denotes the direct channel vector. The summation includes *K* propagation paths, each with a complex gain $\gamma_{k}$. The array response vector $a_{B}\left(\varphi_{k}^{\mathrm{ele}},\varphi_{k}^{\mathrm{azi}}\right)$ models the BS’s response to a signal arriving from the direction specified by the elevation angle $\varphi_{k}^{\mathrm{ele}}$ and azimuth angle $\varphi_{k}^{\mathrm{azi}}$ of the *k*-th path.

The array response of the RIS, denoted by $a_{R}\left(\phi_{l}^{\mathrm{ele}},\phi_{l}^{\mathrm{azi}}\right)$, characterizes how the RIS reflects signals departing at a specific AoD. It is formulated as a vector of complex exponentials, where each element corresponds to a reflecting element on the RIS. The response depends on the elevation angle $\phi_{l}^{\mathrm{ele}}$ and the azimuth angle $\phi_{l}^{\mathrm{azi}}$, while the coordinates $\left(x_{m}^{R},z_{m}^{R}\right)$ of each RIS element determine the corresponding phase shift induced by the signal’s path length. The normalization factor $\frac{1}{\sqrt{M}}$ ensures unit power across the array response vector.

(5) $$\begin{array}{cc} a_{R}\left(\phi_{l}^{\mathrm{ele}},\phi_{l}^{\mathrm{azi}}\right) & =\frac{1}{\sqrt{M}}\left[e^{j\frac{2\pi }{\lambda }\left(\sin\left(\phi_{l}^{\mathrm{ele}}\right)\cos\left(\phi_{l}^{\mathrm{azi}}\right)x_{1}^{R}+\cos\left(\phi_{l}^{\mathrm{ele}}\right)z_{1}^{R}\right)},\ldots ,\right.\\ & \;{\left.e^{j\frac{2\pi }{\lambda }\left(\sin\left(\phi_{l}^{\mathrm{ele}}\right)\cos\left(\phi_{l}^{\mathrm{azi}}\right)x_{M}^{R}+\cos\left(\phi_{l}^{\mathrm{ele}}\right)z_{M}^{R}\right)}\right]}^{T} \end{array}$$

Similarly, the array response of the BS, denoted by $a_{B}\left(\theta_{l}^{\mathrm{ele}},\theta_{l}^{\mathrm{azi}}\right)$, models how the BS receives signals arriving from the user. This response is also represented as a vector of complex exponentials, where the direction of arrival (AoA) is determined by the elevation angle $\theta_{l}^{\mathrm{ele}}$ and azimuth angle $\theta_{l}^{\mathrm{azi}}$. The position of each antenna element at the BS, denoted by $\left(x_{n}^{B},z_{n}^{B}\right)$, affects the signal’s phase shift, and the normalization factor $\frac{1}{\sqrt{N}}$ ensures consistent power normalization.

(6) $$\begin{array}{cc} a_{B}\left(\theta_{l}^{\mathrm{ele}},\theta_{l}^{\mathrm{azi}}\right) & =\frac{1}{\sqrt{N}}\left[e^{j\frac{2\pi }{\lambda }\left(\sin\left(\theta_{l}^{\mathrm{ele}}\right)\cos\left(\theta_{l}^{\mathrm{azi}}\right)x_{1}^{B}+\cos\left(\theta_{l}^{\mathrm{ele}}\right)z_{1}^{B}\right)},\ldots ,\right.\\ \; & \;\;{\left.e^{j\frac{2\pi }{\lambda }\left(\sin\left(\theta_{l}^{\mathrm{ele}}\right)\cos\left(\theta_{l}^{\mathrm{azi}}\right)x_{N}^{B}+\cos\left(\theta_{l}^{\mathrm{ele}}\right)z_{N}^{B}\right)}\right]}^{T} \end{array}$$

The spectral efficiency of the RIS-assisted system is given by the Shannon capacity formula, which is expressed as

(7) $$R=\log_{2}\left(1+\frac{{\left|\left(h_{r}^{H}\Phi G+h_{d}^{H}\right)f\right|}^{2}}{\sigma^{2}}\right),$$

where $\sigma^{2}$ denotes the noise power. The term inside the logarithm represents the signal-to-noise ratio (SNR), which quantifies the strength of the received signal relative to the background noise.

## 3. Problem Formulation

Based on the expression for spectral efficiency in Equation (7), the objective is to maximize the received signal-to-noise ratio (SNR) by jointly optimizing the reflection matrix Φ, the beamforming vector f, and the BS antenna positions x and z:

(8) $$\begin{array}{cc} \underset{\Phi ,f,x,z}{\arg\;\max}\; & {\left|\left(h_{r}^{H}\Phi G+h_{d}^{H}\right)f\right|}^{2}\\ s.t.\; & \left|v_{m}\right|=1,\;\forall m,\\ \; & {∥f∥}_{2}^{2}=P_{t},\\ \; & \sqrt{{\left(x_{i}-x_{j}\right)}^{2}+{\left(z_{i}-z_{j}\right)}^{2}}\geq \frac{\lambda }{2},\;\forall i\neq j. \end{array}$$

This optimization problem is subject to three sets of constraints:

- The unit-modulus constraint on the RIS reflection coefficients $v_{m}$;
- A total transmit power constraint on the precoding vector f;
- A minimum spacing constraint between any two BS antenna elements to prevent spatial correlation.

Since the noise power $\sigma^{2}$ is fixed and uncontrollable, the optimization focuses on maximizing the received signal power. Specifically, maximizing the squared modulus ${\left|\left(h_{r}^{H}\Phi G+h_{d}^{H}\right)f\right|}^{2}$ is equivalent to maximizing the SNR. The modulus squared formulation provides a tractable mathematical representation of the signal power.

Although the objective function is quadratic in f, the overall optimization problem is non-convex due to the coupling between the reflection matrix Φ, the precoder f, and the BS antenna positions. As a result, directly solving this joint problem is intractable. To address this issue, an alternating optimization (AO) strategy is adopted, where one subset of variables is optimized at a time while keeping the others fixed. This iterative procedure is repeated until convergence or a predefined stopping criterion is satisfied.

### 3.1. Optimization of Φ and f

Within the alternating optimization framework, we first focus on optimizing the precoding vector f, assuming that the reflection matrix Φ, the positions of the RIS elements, and the BS antenna positions are fixed. In a multiple-input single-output (MISO) system, maximum ratio transmission (MRT) is an effective precoding strategy that maximizes the received signal power at the user, thereby improving the signal-to-noise ratio (SNR). MRT is optimal in single-user scenarios with perfect channel state information (CSI) and no interference, as it coherently aligns the transmitted signals at the receiver.

According to the MRT principle, the precoding vector should be aligned with the Hermitian transpose of the equivalent channel vector. The optimal precoder is thus given by

(9) $$f=\alpha h_{\mathrm{eq}}^{H},$$

where α is a scalar normalization factor used to satisfy the transmit power constraint, and $h_{\mathrm{eq}}$ denotes the equivalent channel, defined as

(10) $$h_{\mathrm{eq}}=h_{r}^{H}\Phi G+h_{d}^{H}.$$

Substituting $h_{\mathrm{eq}}$ into the precoder expression yields

(11) $$f=\alpha {\left(h_{r}^{H}\Phi G+h_{d}^{H}\right)}^{H}.$$

The transmit power corresponding to f is computed as

(12) $$\begin{array}{cc} {∥f∥}_{2}^{2} & ={∥\alpha {\left(h_{r}^{H}\Phi G+h_{d}^{H}\right)}^{H}∥}_{2}^{2}\\ \; & =\alpha^{2}{∥{\left(h_{r}^{H}\Phi G+h_{d}^{H}\right)}^{H}∥}_{2}^{2}. \end{array}$$

To satisfy the transmit power constraint ${∥f∥}_{2}^{2}=P_{t}$, the scaling factor α is given by

(13) $$\begin{array}{cc} \alpha & =\frac{\sqrt{P_{t}}}{{∥{\left(h_{r}^{H}\Phi G+h_{d}^{H}\right)}^{H}∥}_{2}}. \end{array}$$

Finally, the closed-form expression of the optimal MRT precoder is

(14) $$f=\sqrt{P_{t}}\frac{{\left(h_{r}^{H}\Phi G+h_{d}^{H}\right)}^{H}}{{∥{\left(h_{r}^{H}\Phi G+h_{d}^{H}\right)}^{H}∥}_{2}}.$$

Substituting Equation (14) into the objective function in Equation (8), the problem simplifies to

(15) $${\left|\left(h_{r}^{H}\Phi G+h_{d}^{H}\right)f\right|}^{2}=P_{t}{∥G^{H}\mathrm{diag}\left\{h_{r}^{H}\right\}v+h_{d}∥}_{2}^{2},$$

where $v\in C^{M\times 1}$ is the RIS phase vector with unit-modulus elements.

Expanding the norm squared yields a quadratic form with respect to v:

(16) $${∥G^{H}\mathrm{diag}\left\{h_{r}^{H}\right\}v+h_{d}∥}_{2}^{2}=v^{H}Q_{h}v+v^{H}q_{h}+q_{h}^{H}v+r_{h},$$

where $Q_{h}=\mathrm{diag}\left(h_{r}^{H}\right)GG^{H}\mathrm{diag}\left(h_{r}\right)$, $q_{h}=\mathrm{diag}\left(h_{r}^{H}\right)Gh_{d}$, and $r_{h}=h_{d}^{H}h_{d}$.

To convert the problem into a homogeneous quadratic form, we introduce an auxiliary variable t∈C and define the augmented vector $p={\left[\begin{array}{cc} v^{T} & t \end{array}\right]}^{T}\in C^{M+1}$. Then the objective becomes

(17) $${∥G^{H}\mathrm{diag}\left\{h_{r}^{H}\right\}v+h_{d}∥}_{2}^{2}=p^{H}Qp,$$

where

(18) $$Q=\left[\begin{array}{cc} Q_{h} & q_{h}\\ q_{h}^{H} & 0 \end{array}\right].$$

The problem is then reformulated as a quadratically constrained quadratic program (QCQP):

(19) $$\underset{p\in C^{M+1}}{\arg\;\max}\;p^{H}Qp,\;\mathrm{subject}\;\mathrm{to}\;\left|p_{i}\right|=1,\;\forall i\in \left\{1,\ldots ,M+1\right\}.$$

To solve this non-convex problem under unit-modulus constraints, we adopt an iterative algorithm based on successive projections. At each iteration, the next vector $p^{\left(t+1\right)}$ is selected to minimize the Euclidean distance to $Qp^{\left(t\right)}$, projected onto the unit circle:

(20) $$\min_{p^{\left(t+1\right)}\in \Omega^{M+1}}{∥p^{\left(t+1\right)}-Qp^{\left(t\right)}∥}_{2}.$$

Expanding the squared Euclidean distance yields

(21) $${∥p^{\left(t+1\right)}-Qp^{\left(t\right)}∥}_{2}^{2}=\mathrm{const}-2ℜ\left\{p^{(t+1)H}Qp^{\left(t\right)}\right\},$$

which is equivalent to maximizing

(22) $$\max_{p^{\left(t+1\right)}\in \Omega^{M+1}}ℜ\left\{p^{(t+1)H}Qp^{\left(t\right)}\right\}.$$

Analogous to the traditional power method, we aim to iteratively approximate the dominant eigenvector of Q. In the unconstrained setting, this would lead to the standard update:

(23) $$p^{\left(t+1\right)}=\frac{Qp^{\left(t\right)}}{∥Qp^{\left(t\right)}{∥}_{2}}.$$

However, due to the unit-modulus constraint $|p_{i}|=1$ for all *i*, this update does not satisfy the feasible set. Therefore, we adopt a phase projection strategy, which normalizes each component to have unit magnitude while preserving its phase:

(24) $$p^{\left(t+1\right)}={\left[e^{j\arg({\left[Qp^{\left(t\right)}\right]}_{1})},\ldots ,e^{j\arg({\left[Qp^{\left(t\right)}\right]}_{M+1})}\right]}^{T},$$

thereby preserving the directional information of $Qp^{\left(t\right)}$ while ensuring that each entry of $p^{\left(t+1\right)}$ lies on the complex unit circle. This guarantees feasibility and enables convergence to a locally optimal solution under the unit-modulus constraint.

To establish the convergence behavior of the algorithm, we analyze the monotonicity of the objective function. Let the objective value at iteration *t* be defined as $f^{\left(t\right)}=p^{(t)H}Qp^{\left(t\right)}$. The difference between two successive iterations is given by

(25) $$\begin{array}{cc} f^{\left(t+1\right)}-f^{\left(t\right)} & =p^{(t+1)H}Qp^{\left(t+1\right)}-p^{(t)H}Qp^{\left(t\right)}\\ \; & ={\left(p^{\left(t+1\right)}-p^{\left(t\right)}\right)}^{H}Q\left(p^{\left(t+1\right)}-p^{\left(t\right)}\right)\\ \; & \;+2ℜ\left\{p^{(t+1)H}Qp^{\left(t\right)}\right\}-2ℜ\left\{p^{(t)H}Qp^{\left(t\right)}\right\}. \end{array}$$

Since Q is positive definite, the first term satisfies

(26) $${\left(p^{\left(t+1\right)}-p^{\left(t\right)}\right)}^{H}Q\left(p^{\left(t+1\right)}-p^{\left(t\right)}\right)\geq \sigma_{\min}\left(Q\right){∥p^{\left(t+1\right)}-p^{\left(t\right)}∥}_{2}^{2}\geq 0,$$

where $\sigma_{\min}\left(Q\right)$ denotes the smallest eigenvalue of Q. Moreover, the projection step ensures that

(27) $$ℜ\left\{p^{(t+1)H}Qp^{\left(t\right)}\right\}>ℜ\left\{p^{(t)H}Qp^{\left(t\right)}\right\}.$$

Combining the above inequalities, we conclude that $f^{\left(t+1\right)}>f^{\left(t\right)}$, which confirms that the objective function increases monotonically with each iteration.

### 3.2. Optimization of x,z

The subproblem in (8) with respect to the BS antenna positions x and z can be formulated as

(28) $$\begin{array}{cc} \underset{x,z}{\arg\;\max}\; & {\left|\left(h_{r}^{H}\Phi G+h_{d}^{H}\right)f\right|}^{2}\\ s.t.\; & \sqrt{{\left(x_{i}-x_{j}\right)}^{2}+{\left(z_{i}-z_{j}\right)}^{2}}\geq \frac{\lambda }{2},\;\forall i\neq j. \end{array}$$

The antenna positions x,z directly influence both the cascaded channel and the direct link, and hence impact the received signal power. To analyze this, we first introduce the cascaded channel matrix:

(29) $$\begin{array}{cc} B & =\mathrm{diag}\{h_{r}^{H}\}G\\ \; & =\sum_{l=1}^{L}\sum_{p=1}^{P}\alpha_{p}^{*}\beta_{l}\left(b^{*}\left(\vartheta_{p}^{\mathrm{ele}},\vartheta_{p}^{\mathrm{azi}}\right)⊙b\left(\phi_{l}^{\mathrm{ele}},\phi_{l}^{\mathrm{azi}}\right)\right)a^{H}\left(\theta_{l}^{\mathrm{ele}},\theta_{l}^{\mathrm{azi}}\right)\\ \; & =\sum_{l=1}^{L}\sum_{p=1}^{P}\alpha_{p}^{*}\beta_{l}c\left(\vartheta_{p}^{\mathrm{ele}},\vartheta_{p}^{\mathrm{azi}},\phi_{l}^{\mathrm{ele}},\phi_{l}^{\mathrm{azi}}\right)a^{H}\left(\theta_{l}^{\mathrm{ele}},\theta_{l}^{\mathrm{azi}}\right), \end{array}$$

where the cascaded array response is defined as

(30) $$c\left(\vartheta_{p}^{\mathrm{ele}},\vartheta_{p}^{\mathrm{azi}},\phi_{l}^{\mathrm{ele}},\phi_{l}^{\mathrm{azi}}\right)=b^{*}\left(\vartheta_{p}^{\mathrm{ele}},\vartheta_{p}^{\mathrm{azi}}\right)⊙b\left(\phi_{l}^{\mathrm{ele}},\phi_{l}^{\mathrm{azi}}\right).$$

Left-multiplying the RIS phase vector v, the cascaded channel vector becomes

(31) $$\begin{array}{c} h_{b}^{H}=v^{T}B=\sum_{l=1}^{L}\sum_{p=1}^{P}\eta_{l,p}a^{H}\left(\theta_{l}^{\mathrm{ele}},\theta_{l}^{\mathrm{azi}}\right), \end{array}$$

where $\eta_{l,p}=\alpha_{p}^{*}\beta_{l}v^{T}c\left(\cdot \right)$ is the effective gain of the (l,p)-th cascaded path. Since the array response vector depends only on the index *l*, we can further simplify:

(32) $$h_{b}^{H}=\sum_{l=1}^{L}{\tilde{\eta }}_{l}a^{H}\left(\theta_{l}^{\mathrm{ele}},\theta_{l}^{\mathrm{azi}}\right),$$

where ${\tilde{\eta }}_{l}=\sum_{p=1}^{P}\eta_{l,p}$ represents the aggregated gain of the *l*-th equivalent path.

This reveals that the cascaded channel $h_{b}$ shares the same angular-domain structure as the direct channel $h_{d}$. Therefore, the total equivalent channel can be written as

(33) $$\begin{array}{cc} {\bar{h}}^{H} & =h_{b}^{H}+h_{d}^{H}\\ & =\sum_{l=1}^{L}{\tilde{\eta }}_{l}a^{H}\left(\theta_{l}^{\mathrm{ele}},\theta_{l}^{\mathrm{azi}}\right)+\sum_{k=1}^{K}\gamma_{k}^{*}a^{H}\left(\varphi_{k}^{\mathrm{ele}},\varphi_{k}^{\mathrm{azi}}\right). \end{array}$$

Since both terms in (33) share the same array response structure, we merge them into a unified angular-domain representation. To this end, we define a consolidated parameter set

$$\Omega =\{{\tilde{\eta }}_{l},\gamma_{k},\theta_{l}^{\mathrm{ele}},\theta_{l}^{\mathrm{azi}},\varphi_{k}^{\mathrm{ele}},\varphi_{k}^{\mathrm{azi}}|\forall l,k\}.$$

For notational convenience, we reindex all paths and define $\xi_{\ell }$ as the complex path gain, and $\nu_{\ell }^{\mathrm{ele}}$ and $\nu_{\ell }^{\mathrm{azi}}$ as the corresponding elevation and azimuth angles of the *ℓ*-th path. The equivalent channel vector is then expressed as

(34) $${\bar{h}}^{H}=\sum_{\ell =1}^{K+L}\xi_{\ell }a^{H}\left(\nu_{\ell }^{\mathrm{ele}},\nu_{\ell }^{\mathrm{azi}}\right),$$

where $\xi_{\ell },\nu_{\ell }^{\mathrm{ele}},\nu_{\ell }^{\mathrm{azi}}\in \Omega$.

Accordingly, the objective function in (8) can now be rewritten as $|{\bar{h}}^{H}{\left(x,z\right)f|}^{2}$, and the optimization problem is reformulated as

(35) $$\begin{array}{cc} \underset{x,z}{\arg\;\max}\; & {\left|{\bar{h}}^{H}\left(x,z\right)f\right|}^{2}\\ \;s.t. & \sqrt{{\left(x_{i}-x_{j}\right)}^{2}+{\left(z_{i}-z_{j}\right)}^{2}}\geq \frac{\lambda }{2},\;\forall i\neq j. \end{array}$$

This is a constrained nonlinear optimization problem, where the objective is non-convex and the constraints are nonlinear. To address this, we adopt the SQP method [39], which iteratively solves a series of QP subproblems that approximate the original problem. At each iteration *t*, the SQP algorithm executes the following steps:

- Approximates the original problem by a quadratic model of the Lagrangian;
- Linearizes the nonlinear constraints;
- Solves the resulting QP to obtain a search direction;
- Performs a line search to determine an appropriate step size;
- Updates the current solution.

The core idea is to emulate Newton’s method for constrained optimization by approximating and solving the Karush–Kuhn–Tucker (KKT) conditions at each iteration.

We first reformulate the original problem into a minimization problem and rewrite the constraints in a standard form:

(36) $$\begin{array}{cc} \underset{x,z}{\arg\;\min}\; & f(x,z)\\ s.t.\; & g_{ij}\left(x,z\right)\leq 0,\;\forall i\neq j, \end{array}$$

where the objective function is defined as $f\left(x,z\right)=-{\left|{\bar{h}}^{H}\left(x,z\right)f\right|}^{2}$, and the constraint functions are given by

(37) $$g_{ij}\left(x,z\right)=\frac{\lambda }{2}-\sqrt{{\left(x_{i}-x_{j}\right)}^{2}+{\left(z_{i}-z_{j}\right)}^{2}}.$$

The Lagrangian function that combines the objective and constraints is constructed as

(38) $$L\left(x,z,\lambda \right)=f\left(x,z\right)+\sum_{i<j}\lambda_{ij}g_{ij}\left(x,z\right),$$

where $\lambda_{ij}>0$ are the Lagrange multipliers associated with each inter-antenna distance constraint. This formulation transforms the constrained problem into an unconstrained one by penalizing violations of the constraints.

The optimal solution to the Lagrangian formulation must achieve a balance between minimizing the objective function and satisfying the spatial separation constraints. To this end, we employ a second-order Taylor approximation of the Lagrangian, which captures local curvature information via second derivatives. This approach is analogous to Newton’s method, but adapted for constrained problems via SQP.

Let $\chi ={\left[x^{T},z^{T}\right]}^{T}\in R^{2N}$ denote the concatenated vector of BS antenna positions. At iteration *t*, with current iterate $\chi_{t}$, the Lagrangian function is approximated by a second-order Taylor expansion as

(39) $$L\left(\chi_{t}+d,\lambda_{t}\right)\approx L\left(\chi_{t},\lambda_{t}\right)+\nabla L{\left(\chi_{t},\lambda_{t}\right)}^{T}d+\frac{1}{2}d^{T}\nabla^{2}L\left(\chi_{t},\lambda_{t}\right)d,$$

where $d\in R^{2N}$ is the search direction, $\lambda_{t}$ denotes the Lagrange multipliers at iteration *t*, and ∇L and $\nabla^{2}L$ represent the gradient and Hessian of the Lagrangian, respectively.

The constraints are linearized around $\chi_{t}$ to obtain a first-order approximation:

(40) $$g_{ij}\left(\chi_{t}+d\right)\approx g_{ij}\left(\chi_{t}\right)+\nabla g_{ij}{\left(\chi_{t}\right)}^{T}d\leq 0,$$

which maintains the convexity of the resulting QP subproblem and provides a locally accurate feasible region.

Based on the above approximations, the quadratic programming subproblem at iteration *t* is formulated as

(41) $$\begin{array}{cc} \underset{d}{\arg\;\min}\; & \frac{1}{2}d^{T}U_{t}d+\nabla f{\left(\chi_{t}\right)}^{T}d\\ s.t.\; & \nabla g_{ij}{\left(\chi_{t}\right)}^{T}d\leq g_{ij}\left(\chi_{t}\right),\;\forall i\neq j, \end{array}$$

where $U_{t}$ is a positive definite approximation of the Hessian $\nabla^{2}L\left(\chi_{t},\lambda_{t}\right)$, which can be efficiently updated using quasi-Newton methods:

(42) $$U_{t+1}=U_{t}-\frac{U_{t}m_{t}m_{t}^{T}U_{t}}{m_{t}^{T}U_{t}m_{t}}+\frac{r_{t}r_{t}^{T}}{m_{t}^{T}r_{t}},$$

where $m_{t}=\chi_{t+1}-\chi_{t}$ and $r_{t}=\nabla L\left(\chi_{t+1},\lambda_{t+1}\right)-\nabla L\left(\chi_{t},\lambda_{t+1}\right)$.

The overall steps of the proposed SQP-based antenna position optimization are summarized in Algorithm 1, which provides a structured description of the iterative solution procedure.
**Algorithm 1** SQP for Antenna Position Optimization1:**Initial:** position $\chi_{0}={\left[x_{0}^{T},z_{0}^{T}\right]}^{T}$, hessian approximation $U_{0}=I_{2N\times 2N}$, lagrange multipliers $\lambda_{0}=0$, tolerance ϵ>0, penalty parameter μ>02:Set t←03:**while**$\;∥\chi_{t+1}-\chi_{t}{∥}_{2}\;\geq \epsilon \;$**or**$\max g_{ij}\left(\chi_{t+1}\right)>\epsilon \;$**do**4:    Compute $\nabla f(\chi_{t})$ and $\nabla g_{ij}\left(\chi_{t}\right)$5:    Solve the QP:$$\begin{array}{cc} \min_{d} & \;\frac{1}{2}d^{T}U_{t}d+\nabla f{\left(\chi_{t}\right)}^{T}d\\ s.t. & \;\nabla g_{ij}{\left(\chi_{t}\right)}^{T}d\leq -g_{ij}\left(\chi_{t}\right),\;\forall i<j \end{array}$$6:    Obtain search direction $d_{t}$ and multipliers $\lambda_{t+1}$7:    Find step size $\alpha_{t}\in \left(0,1\right]$ minimizing the merit function:$$\begin{array}{c} \phi \left(\alpha \right)=f\left(\chi_{t}+\alpha d_{t}\right)+\mu \sum_{i<j}\max\left(0,g_{ij}\left(\chi_{t}+\alpha d_{t}\right)\right) \end{array}$$8:    $\chi_{t+1}\leftarrow \chi_{t}+\alpha_{t}d_{t}$9:    $m_{t}=\chi_{t+1}-\chi_{t}$10:   $r_{t}\leftarrow \nabla_{\chi }L\left(\chi_{t+1},\lambda_{t+1}\right)-\nabla_{\chi }L\left(\chi_{t},\lambda_{t+1}\right)$11:   Update $U_{t+1}$ via BFGS:$$\begin{array}{c} U_{t+1}=U_{t}-\frac{U_{t}m_{t}m_{t}^{T}U_{t}}{m_{t}^{T}U_{t}m_{t}}+\frac{r_{t}r_{t}^{T}}{m_{t}^{T}r_{t}} \end{array}$$12:    t←t+113:**end while**14:**Return: **$\chi^{*}\leftarrow \chi_{t}$

## 4. Results

This section presents numerical simulations to evaluate the spectral efficiency performance of the proposed optimization framework. The system operates at a carrier frequency of 10 GHz. The BS and RIS are equipped with N=4 and M=4 antennas, respectively. The number of propagation paths is set as L=P, and the line-of-sight path exists. The inter-element spacing at the RIS is fixed to λ/2. The user and scatterers are randomly positioned, with elevation angles uniformly drawn from [0,π] and azimuth angles from [0,2π]. The BS antennas are confined within a square region of side length *R*, referred to as the movable region.

**Figure 2:**
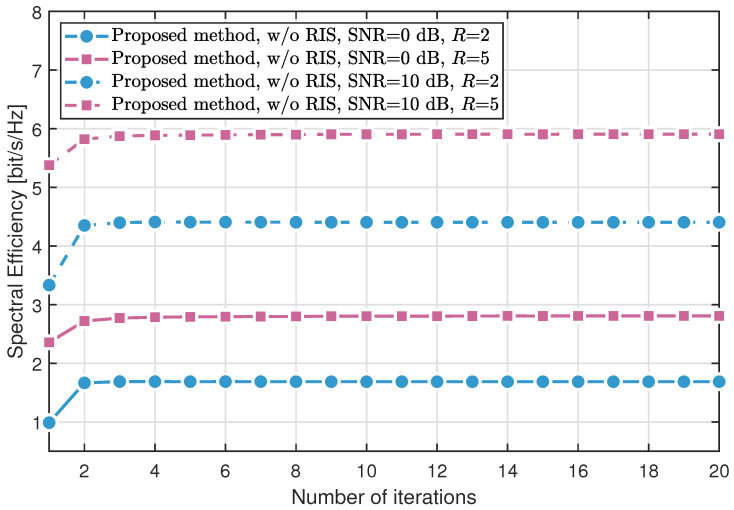
Spectral efficiency versus the number of iterations without RIS.

**Figure 3:**
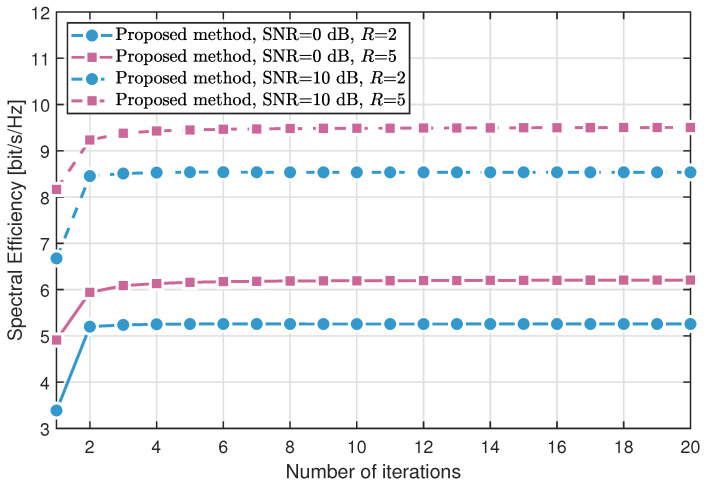
Spectral efficiency versus the number of iterations with RIS.

Figure 2 and Figure 3 compare the convergence behavior of the proposed method in two configurations: without RIS assistance and with RIS assistance, respectively. The simulations are conducted under L=P=10 multipath components, signal-to-noise ratios (SNRs) of 0 dB and 10 dB, and movable region sizes R∈{2,5} meters. The results show that the spectral efficiency increases with the number of iterations and converges within just three iterations, demonstrating the rapid convergence of the algorithm. Moreover, spectral efficiency is significantly influenced by both the SNR and the size of the movable region.

**Figure 4:**
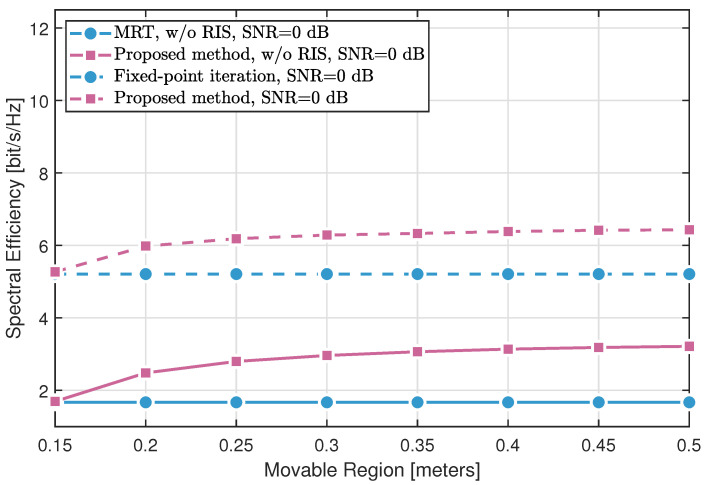
Spectral efficiency of different methods varying with the movable size (SNR = 0 dB).

**Figure 5:**
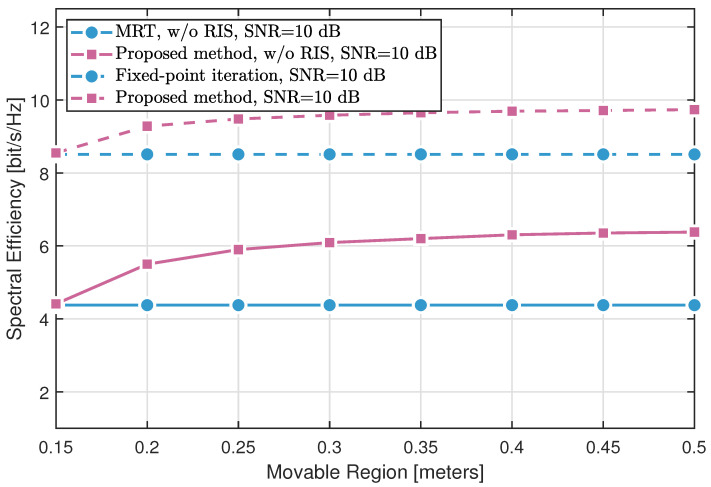
Spectral efficiency of different methods varying with the movable size (SNR = 10 dB).

Figure 4 and Figure 5 demonstrate the impact of the movable region size on the spectral efficiency across different methods. Three key observations can be made:

- The fixed-point iteration method with RIS assistance significantly outperforms conventional MRT with fixed BS antennas, validating the effectiveness of RIS in enhancing the propagation environment.
- The proposed MA optimization approach without RIS yields notable gains over MRT by exploiting antenna position diversity through SQP-based optimization. When combined with RIS, the proposed method further surpasses the fixed-point RIS design, highlighting the synergistic benefits of joint MA and RIS optimization.
- Comparing the results at different SNR levels (0 dB in Figure 4 and 10 dB in Figure 5) reveals that higher SNR amplifies the advantages offered by both MA and RIS.

In summary, both RIS deployment and SQP-based antenna position optimization independently enhance spectral efficiency, while their combination achieves the most significant performance improvement.

**Figure 6:**
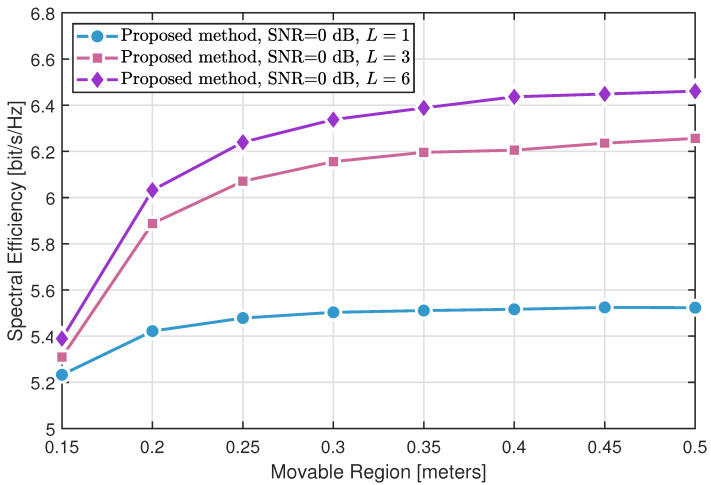
Spectral efficiency of the proposed method varying with *L* channel paths (SNR = 0 dB).

**Figure 7:**
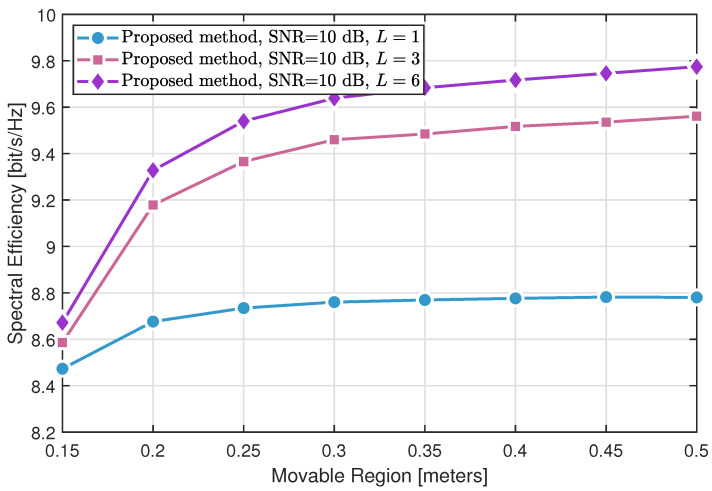
Spectral efficiency of the proposed method varying with *L* channel paths (SNR = 10 dB).

Figure 6 and Figure 7 illustrate the critical role of multipath richness in enhancing the spectral efficiency of the proposed method. While only marginal improvements are observed under sparse scattering conditions (L=1), significant performance gains are achieved in richer multipath environments (L=6 and L=9), especially when combined with larger movable regions. This improvement is attributed to the MA system’s ability to exploit constructive multipath interference through optimized antenna positioning.

However, as the movable region size continues to increase, the performance gain gradually saturates, revealing an inherent trade-off between spectral efficiency and antenna mobility complexity. These findings quantitatively confirm that the proposed approach delivers the most pronounced benefits when both sufficient multipath diversity and adequate antenna mobility are available.

**Figure 8:**
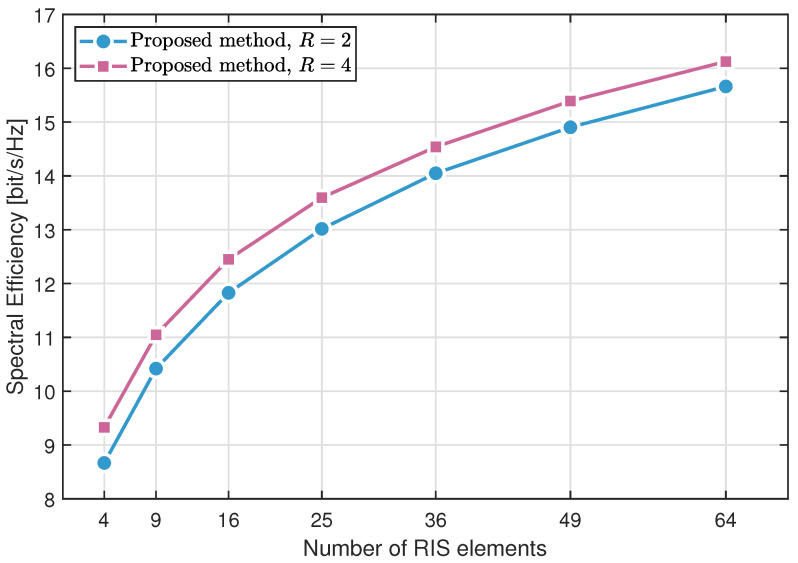
Spectral efficiency varying with the number of RIS elements.

In Figure 8, we evaluate the impact of the number of RIS elements on the performance. As the number of RIS elements increases, the system performance improves in a nonlinear manner.

## 5. Conclusions

Recognizing the potential of RIS and MA to enhance channel conditions, this paper investigates their joint deployment for improving wireless communication performance. Specifically, we consider RIS-aided MA systems and propose an alternating optimization framework that jointly optimizes:

- BS beamforming via MRT;
- RIS beamforming via fixed-point iteration;
- BS antenna positions via SQP.

In particular, the SQP method effectively addresses the non-convex antenna position optimization problem by solving a sequence of quadratic programming subproblems under spatial separation constraints. Simulation results demonstrate that the proposed framework fully exploits the complementary characteristics of RIS and MA, and achieves significant spectral efficiency gains through their synergistic integration. This paper assumes perfect channel state information (CSI) as well as ideal antenna movement. In future work, we plan to extend our analysis to more practical scenarios, including imperfect CSI and constrained antenna mobility.

## Data Availability

The raw data supporting the conclusions of this article will be made available by the authors on request.
